# Research trends in the mental health and multimorbidity of older people from 2002 to 2022: A bibliometric analysis *via* CiteSpace

**DOI:** 10.3389/fpsyt.2023.1117554

**Published:** 2023-03-07

**Authors:** Jie Zhou, Dan Song, Juanjuan Ma, Guowen Zhang, Chuduo Wu, Qian Chen, Li Zeng

**Affiliations:** ^1^Department of Nursing, Shenzhen Qianhai Shekou Free Trade Zone Hospital, Shenzhen, Guangdong, China; ^2^The First Affiliated Hospital of USTC, Anhui Provincial Hospital, Hefei, Anhui, China

**Keywords:** multimorbidity, old adults, mental health, bibliometric analysis, research trends

## Abstract

**Purpose:**

Using bibliometric analysis, the objective of this study was to identify research hotspots and trends on multimorbidity and mental health in older adults. This could help guide future research on this topic.

**Methods:**

We searched the Web of Science Core Collection for eligible studies. Type of publications were not restricted and the timeframe was set between 2002 and 2022. Knowledge maps were created using CiteSpace to visualize publications, nations, journals, institutions, authors, cited references, and keywords. Microsoft Excel displayed pertinent tables.

**Results:**

A total of 216 studies were collected for analysis. The annual publication over the past 20 years demonstrated an upward trend. The main contributors in publications were in North America, Europe, Asia, and Oceania, of which aging was a primary issue. However, collaboration between countries, institutions, and authors were relatively sparce. Cluster analysis and co-citation analysis of references and keywords revealed that the research field could be subdivided into four themes: social psychology as the fundamental disciplinary base, Prevalence of mental disorders and multimorbidity in older adults, pertinent health conditions, and effective interventions. Research trends at present lies in health status, risk factors of prognoses, and effective interventions for prevention and management.

**Conclusion:**

The results revealed a reciprocal risk relationship between mental health and multimorbidity. Mental disorders such as depression and anxiety in older adults with multimorbidity garnered considerable interest in the defined population, and further exploration is still promising. Substantial studies on evidence-based prevention and treatment strategies are warranted for improved prognoses.

## Introduction

Multimorbidity commonly refers to an individual's coexistence of at least two chronic conditions (1). Occurred a few decades earlier, multimorbidity has rapidly become a major global challenge due to its substantial effects (2). As more than 70% of inpatient and outpatient services were used by multimorbidity, healthcare systems suffered from critical challenges and pressure (3). Meanwhile, multimorbidity is significantly progressive with age. A prevalence of 91.8% for multimorbidity among adults aged over 65 years was reported in the United States (4). Chen et al. (3) also pointed out that the prevalence of multimorbidity increased from 54.9 to 60.6% between those aged 60–69 years and over 70 years in China. Since various chronic conditions simultaneously develop with age, multimorbidity in older people may induce worsened healthcare outcomes such as prolonged hospital stay, more frequent readmission, premature death, poorer quality of life, and extra cost (5, 6).

Also, Multimorbidity was proven closely associated with mental health. A meta-analysis across 43 low and middle-income countries showed a significant relationship between depression and multimorbidity, with exceptionally high odds ratios in China (Odds Ratio = 8.84) (7). Moreover, those with multimorbidity were two to three times more likely to develop depressive disorders than those without any chronic physical condition, and each additional chronic disease increased the risk by 45% (8). In addition, mental disorders impede patients from seeking treatments, making multimorbidity management more complex and challenging. Furthermore, A cross-sectional study involving over six low and middle-income countries highlighted increased risks among the elderly with multimorbidity and anxiety (9).

Although several reviews explored multimorbidity or mental health in old adults, limited research interest has focused on both of them (10, 11). Previous studies mainly attempt to explore their prevalence, impacts and prognostics. Whereas available interventions and relevant effects on prevention and management are under-explored. The most crucial point is a deficiency of objectively providing a comprehensive picture of this research field. Unlike traditional analytic methods, bibliometric analysis refers to quantitative and statistical workflow clarifying the scientific knowledge structure of specific fields (12). The application of bibliometrics ensures analysis of vast amounts of publications and patterns on microscopic and macroscopic levels (13). More significantly, it can shed light on the evolution of the research field and assist researchers in identifying the prospective research field's evolutionary nuances and research trends (14). Thus, we performed a bibliometric analysis of available publications on mental health and multimorbidity in older people using the CiteSpace tool from 2002 to 2022. Accordingly, an overview of the current research status and relevant research trends and hotspots in the field are reported. Specifically, the research questions were as follows:

1) What are the most influential journals and disciplines in the field?

2) Which countries, institutions, authors contributed the most to the field?

3) What are the main research trends and hotspots in this field?

## Methods

### Data collection

Bibliometric analysis is based on literature databases, such as Web of Science, Scopus, PubMed, Embase, et al. Containing numerous international high-impact academic journals, the Web of Science database is proven to provide better knowledge map effect for visual analysis using CiteSpace (15). Therefore, it is reasonable and practical to adopt the Web of Science as the data source. Specifically, we searched the Web of Science Core Collection (WoSCC), including all Editions. To comprehensively investigate the research development in this field and avoid bias due to daily updates, publications are retrieved on January 3, 2023, with the timeframe from January 1, 2002, to December 3, 2022.

The search strategy is built as follows: TI = (old population OR old^*^ person OR old^*^ people OR old^*^ adult OR old^*^ man OR old^*^ woman OR elder^*^ OR aging OR geriatric^*^ Or senior) AND TS = (multimorbidit^*^ OR multi-morbidit^*^ OR multi morbidit^*^ OR multiple morbidities OR multiple-morbidities OR comorbidit^*^ OR Polymorbidit^*^ OR multiple chronic disease^*^ OR multiple chronic condition^*^ OR ((multiple OR coexisting OR co-existing OR concurrent OR con-current OR comorbid OR co-morbid) And (disease^*^ OR illness^*^ OR condition^*^ OR diagnos^*^ OR morbid^*^))) AND TS = (((mental OR psychological) AND (health OR wellbeing OR well-being OR illness OR disease^*^ OR disorder^*^)) OR depress^*^ OR anxiety). Detailed search history is presented in Supplementary Figure 1. There are no restrictions for publication language and literature type. To ensure the quality of included data, an initial screen of the records is performed according to the predefined exclusion criteria: (1) the topic is not about multimorbidity in older adults; (2) the topic is not about mental health; (3) the main research object is not the older people or involved other population; (4) repeated literature. Finally, 216 references are exported in plain text format with full records cited references and furtherly saved as “download_^*^” in a new folder “input.” The “^*^” represents a sequential number. Additional folders are created as “output,” “data,” and “project.” CiteSpace (version 6.1.R6) is used for data format conversion and deduplication, which resulted in a set of “download ^*^.txt” documents in the folder “output.” After copying them to the “data” folder, knowledge maps for bibliometric visualization analysis are generated with CiteSpace by applying specific modes and parameters.

### Data analysis

Currently, there are several research tools for bibliometric analysis such as VOSviewer, Biblioshiny or CiteSpace, of which specific advantages and shortcomings are both existed. This research selected CiteSpace considering its interactive visualization and optional functions like data mining algorithms (16). Detailed parameters were set as follows: time slicing was set from 2002 JAN to 2022 DEC with 1 Year Per Slice, and term source options were applied. The threshold was selected at the top 100 levels of the most cited or occurred items from each slice (Top = 100). Knowledge maps with nodes and lines complete CiteSpace visual analysis. Authors, institutions, and keywords are represented as nodes, which become thicker as their frequency increases. Lines connect nodes, and their thickness indicates their strength. Network analysis relies on nodes with greater centrality (0.1). Knowledge maps provide general features in the top left corner. When the network's modularity (Q value) exceeds 0.3, it indicates a large internal structure. The mean silhouette (S value) verifies cluster internal consistency, showing that clustering findings are acceptable (S value >0.5) and dependable (S value >0.7) (17).

## Results

### Basic statistical analysis

**Figure 1:**
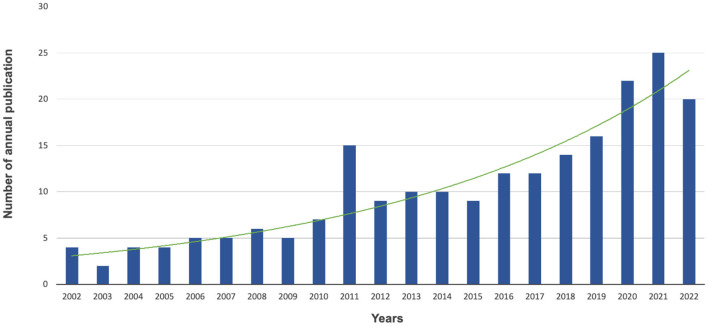
Statistics and trends of annual publications from 2002 to 2022.

Although research on mental health and multimorbidity separately occurred early, publications focused on both of them in older people were scarce before 2004. Subsequently, relevant studies continuously increased and first peaked in 2011. Following a slight fluctuation between 2012 and 2016, the trend kept increasing and peaked again in 2021 as shown in Figure 1. Overall, relatively limited research interest was concentrated on mental health and multimorbidity in older people, and the annual number of relevant publications showed a tendency to grow.

### Journal analysis

A total of 216 records were retrieved from 117 journals. Table 1 lists the top ten journals. Journal of Affective Disorders provided the most publications. American Journal of Geriatric Psychiatry and European Psychiatry also published many studies. Journals were mainly related to psychiatry and geriatrics. Most journals were published in England and the United States. Four journals' impact factor exceeded 7, whereas the average was worked out roundly at 5.71.

**Table 1:** Summary of the publications of the top ten journals.

**Journal**	***N* (%)**	**Country**	**Impact factor (2022)**
Journal of Affective Disorders	11 (5.10)	Netherlands	6.533
American Journal of Geriatric Psychiatry	10 (4.63)	United States	7.996
European Psychiatry	9 (4.17)	England	7.156
International Journal of Geriatric Psychiatry	8 (1.91)	England	3.850
International Psychogeriatrics	8 (3.70)	England	7.191
Journal of the American Geriatrics Society	8 (3.71)	England	7.538
Gerontologist	8 (3.72)	United States	5.422
PLoS ONE	7 (3.24)	United States	3.752
Aging Mental Health	6 (2.78)	England	3.514
BMC Psychiatry	6 (2.79)	England	4.144

### Category analysis

**Figure 2:**
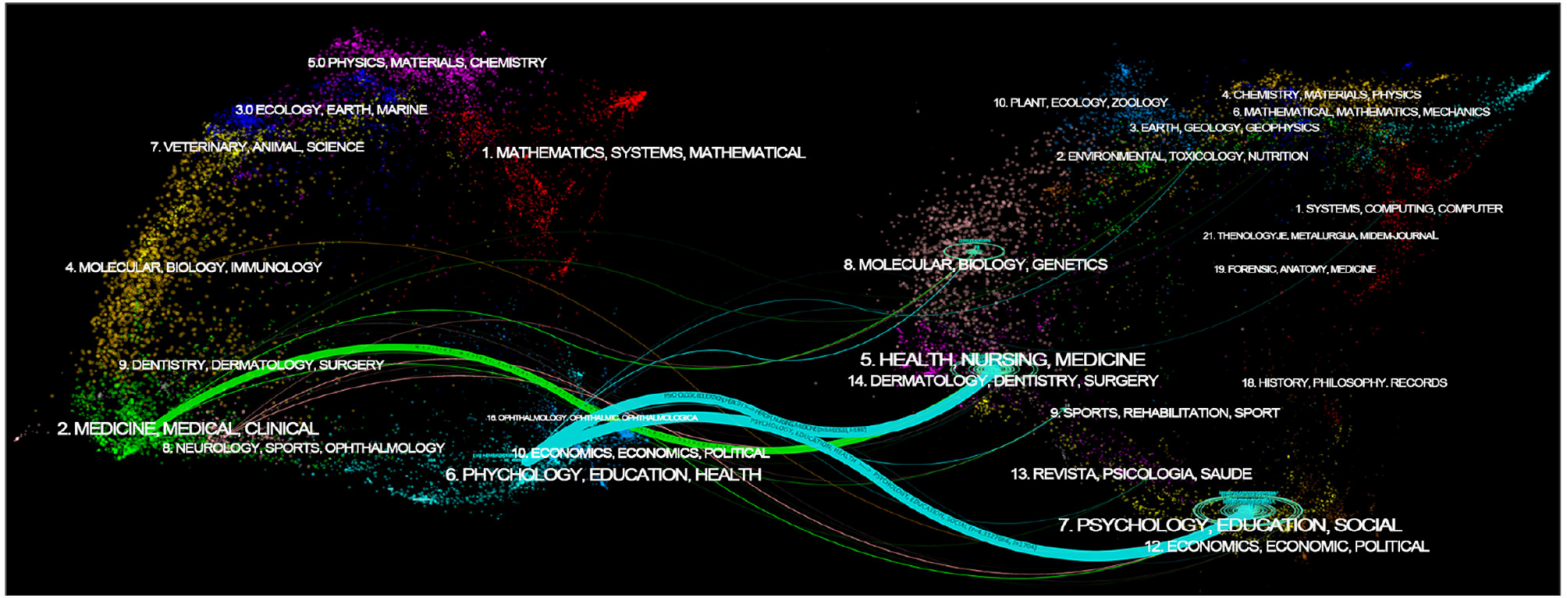
Dual map overlap of journals on the mental health and multimorbidity in older people.

Dual-map overlays of journals are knowledge carriers demonstrating interdisciplinary interaction and collaboration (18). Within a dual-map overlay, the citing journals are on the left while the cited journals are on the right; the colored lines represent the citation relationship. By adjusting the Z score, lines were merged to display more visible citation paths, of which bigger circular ripples indicated more cited journals (19). As per dual-map overlays on mental health and multimorbidity in older people shown in Figure 2, journals were primarily focused on two discipline clusters, namely *Medicine, Medical, Clinical* and *Psychology, Education, Health*. In addition, *Neurology, Sports, Ophthalmology* and *Economics, Nursing*, Political, and other disciplines were also involved in certain degrees. Three notable citation paths originated from *Psychology, Education, Health Medicine, Medical, and Clinical*, which are accordingly referred to *Health, Nursing, Medicine* and *psychology, education, social*. This indicated that the disciplinary basis in this field mainly lies in Psychology and Medicine.

### Co-country analysis

**Figure 3:**
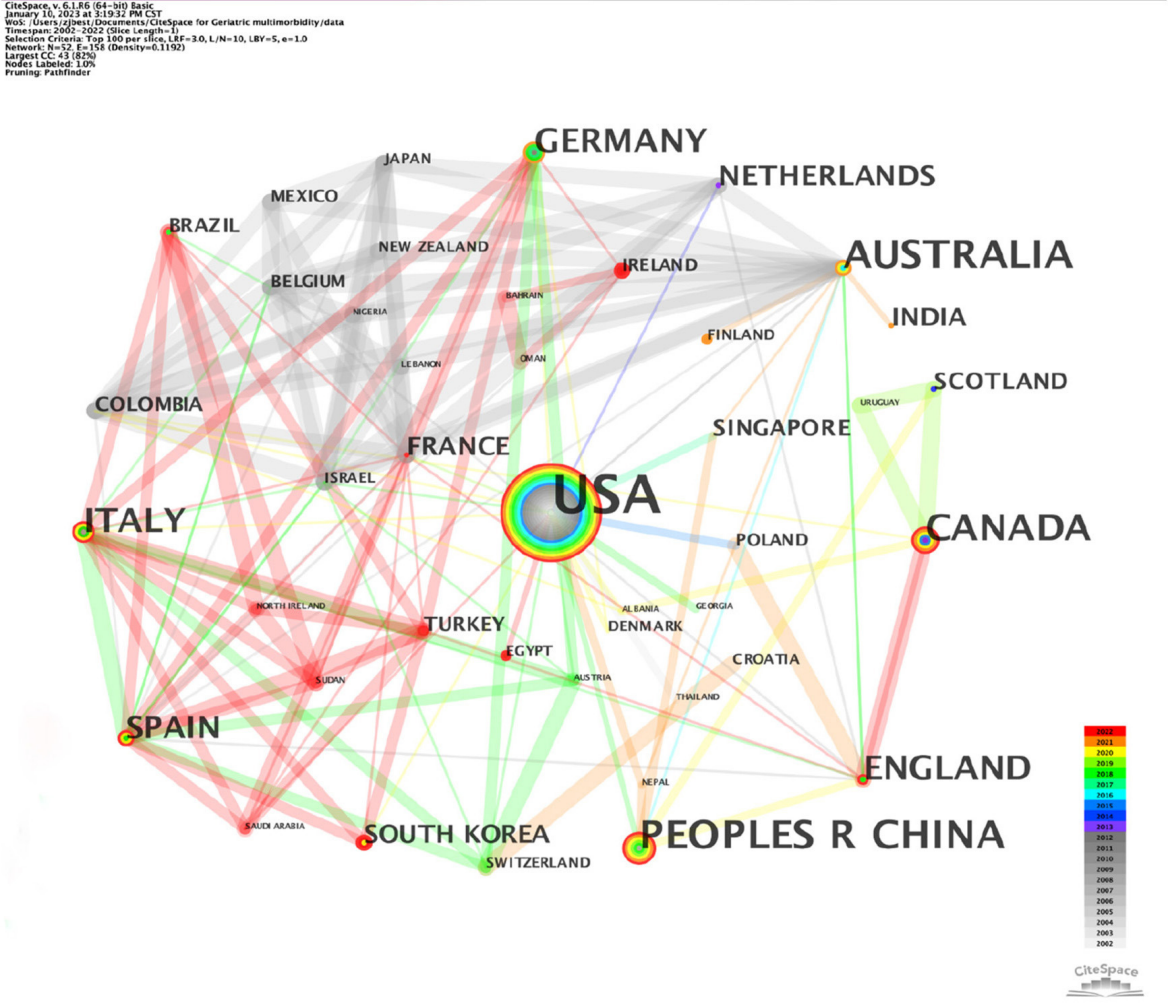
A country collaboration map.

Figure 3 depicts the country distribution, which consists of 52 nodes and 158 linkages, based on the TOP *N* = 100 levels of most cited or occurred items from each slice. The nodes represent countries, which are linked with lines displaying collaboration relationships. Guided by Chen (17), increasing publications will enlarge nodes' size; equally, links become thicker as the relationship between nodes is strengthened.

In total, 52 countries released 216 records. The United States showed the highest frequency of occurrence (86) and centrality (0.41), which indicated that it was the most influential in the collaboration network, followed by China (20), Australia (21), Canada (22), and England (17) as shown in Table 2. The United States reported three times greater frequency than China, implying that geriatric multimorbidity and mental health would be a substantial public health problem there. This was consistent with Boersma's (23) finding that more than half of US people suffered from chronic diseases and that 27.2% had several chronic disorders. In addition, the top five countries by centrality were the United States (0.41), Australia (0.21), France (0.16), Ireland (0.13), and Spain (0.13). Since their centralities were more significant than 0.10, they are believed to impact the research field significantly.

**Table 2:** The top five countries related to geriatric multimorbidity and mental health.

**Ranking**	**Frequence**	**Country**	**Ranking**	**Centrality**	**Country**
1	86	United States	1	0.41	United States
2	24	China	2	0.21	Australia
3	21	Australia	3	0.16	France
4	20	Canada	4	0.13	Ireland
5	15	England	5	0.13	Spain

### Distribution of institutions

**Figure 4:**
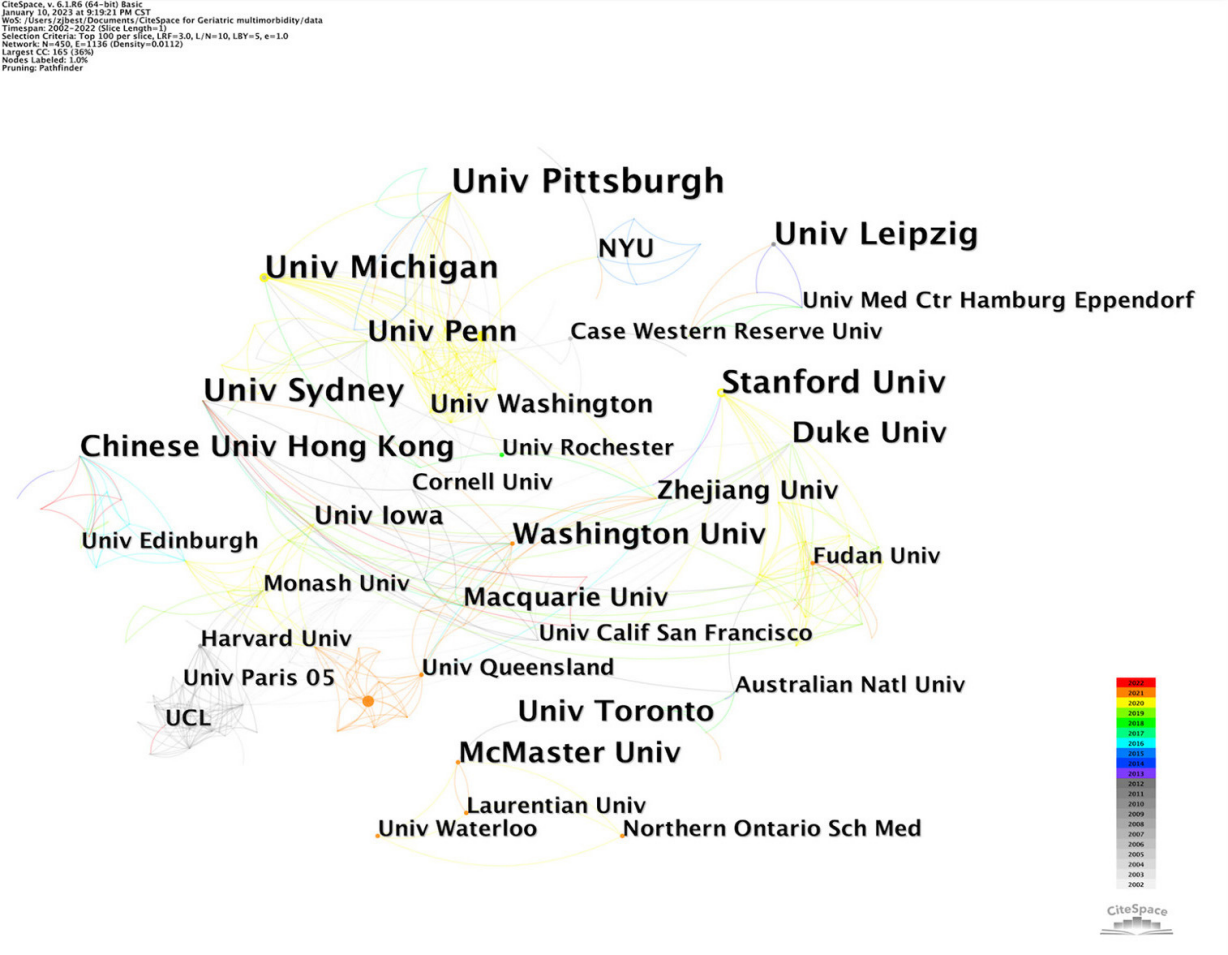
An institution collaboration map.

Figure 4 shows the collaboration relationship between institutions in this research field of mental health and multimorbidity in older adults. Table 3 shows the detailed top five publishing institutions. The University of Pittsburgh topped the list in frequency (United States; seven times), followed by Stanford University (United States; six times), University of Michigan (United States; six times), University of Sydney (Australia; six times), and University of Leipzig (Germany; six times). In general, universities hosted the majority of research institutes, and research collaboration between institutions was limited.

**Table 3:** The top five institutions related to geriatric multimorbidity and mental health.

**Ranking**	**Institution**	**Frequency**	**Country**	**Year**
1	University of Pittsburgh	7	United States	2002
2	Stanford University	6	United States	2002
3	University of Michigan	6	United States	2006
4	University of Sydney	6	Australia	2011
5	Univeristy of Leipzig	6	Germany	2010

“Year” represents the earliest publication time of the institution in the field.

### Co-author analysis

**Figure 5:**
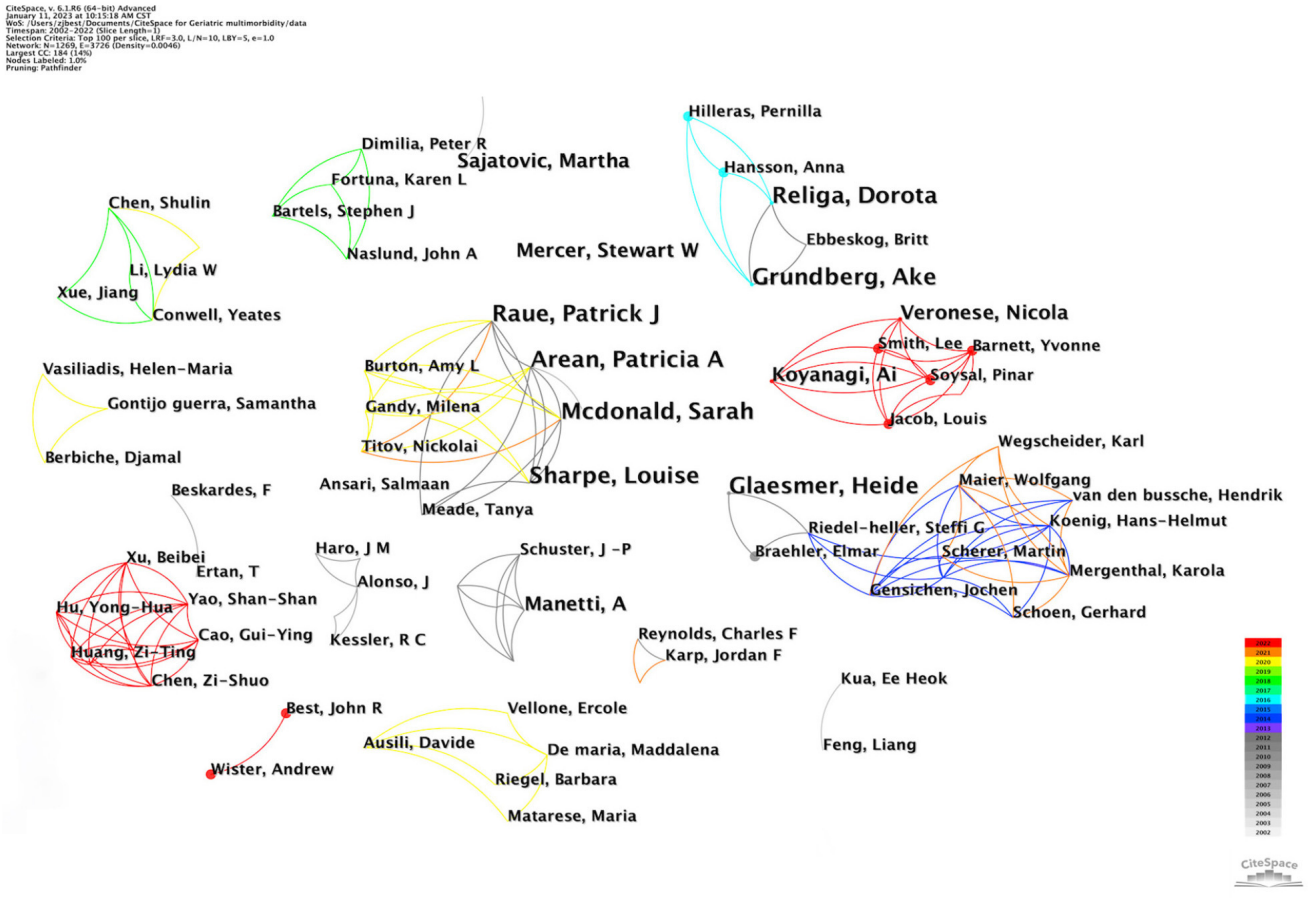
Co-authorships network of authors.

Figure 5 demonstrates a co-authorship network map. Each Circle represents an author, and its size indicates the frequency of their publications. Lines depict the cooperation between authors, with thicker lines indicating greater collaboration. The high-yield scholar was not identified. The network density was 0.0046, which suggested that overall author collaborations were relatively scarce.

Co-citation analysis, which includes author co-citation analysis (ACA), is the most often used method in bibliometric analysis (24, 25). ACA refers to co-citation connections between authors concurrently cited by published research and allows for identifying scholars with the most contribution to a specific field (26). Table 4 displays the top five authors with the highest publication frequency and co-citation. Glaesmer et al. led the publication frequency category. Kessler RC from the Harvard Medical School was the most frequently cited author. The research team in which she was involved pointed out that mental problems increased the likelihood of multimorbidity over a minimum of 15 years, with the 1st year showing the most pronounced influence (22). A comparison of the two groups of the table detected no direct relationship between the frequency of publication and co-citation. In addition, active cooperation was identified among Raue et al.

**Table 4:** The top five authors for publications frequency and co-citations.

**Frequency**	**Author**	**Institution**	**Year**	**Country**
**Base on publication frequency**				
4	Glaesmer, Heide	University of Leipzig	2010	Germany
4	Raue, Patrick J	University of Washington	2012	United States
4	Arean, Patrick	University of Washington	2007	United States
4	Grundberg, Ake	Sophiahemmet University	2012	Sweden
4	Mcdonald, Sarah	University of Sydney	2012	Australia
**Base on co-citation**				
26	Kessler, Ronald C	Harvard University	2006	United States
22	Barnett Karen	University of Dundee	2014	England
18	Read J	University of Sydney	2020	Australia
16	Marengoni A	Karolinska Institutet	2014	Sweden
15	Kroenke K	Mayo Clinic	2005	United States

### Analysis of co-citation references

**Figure 6:**
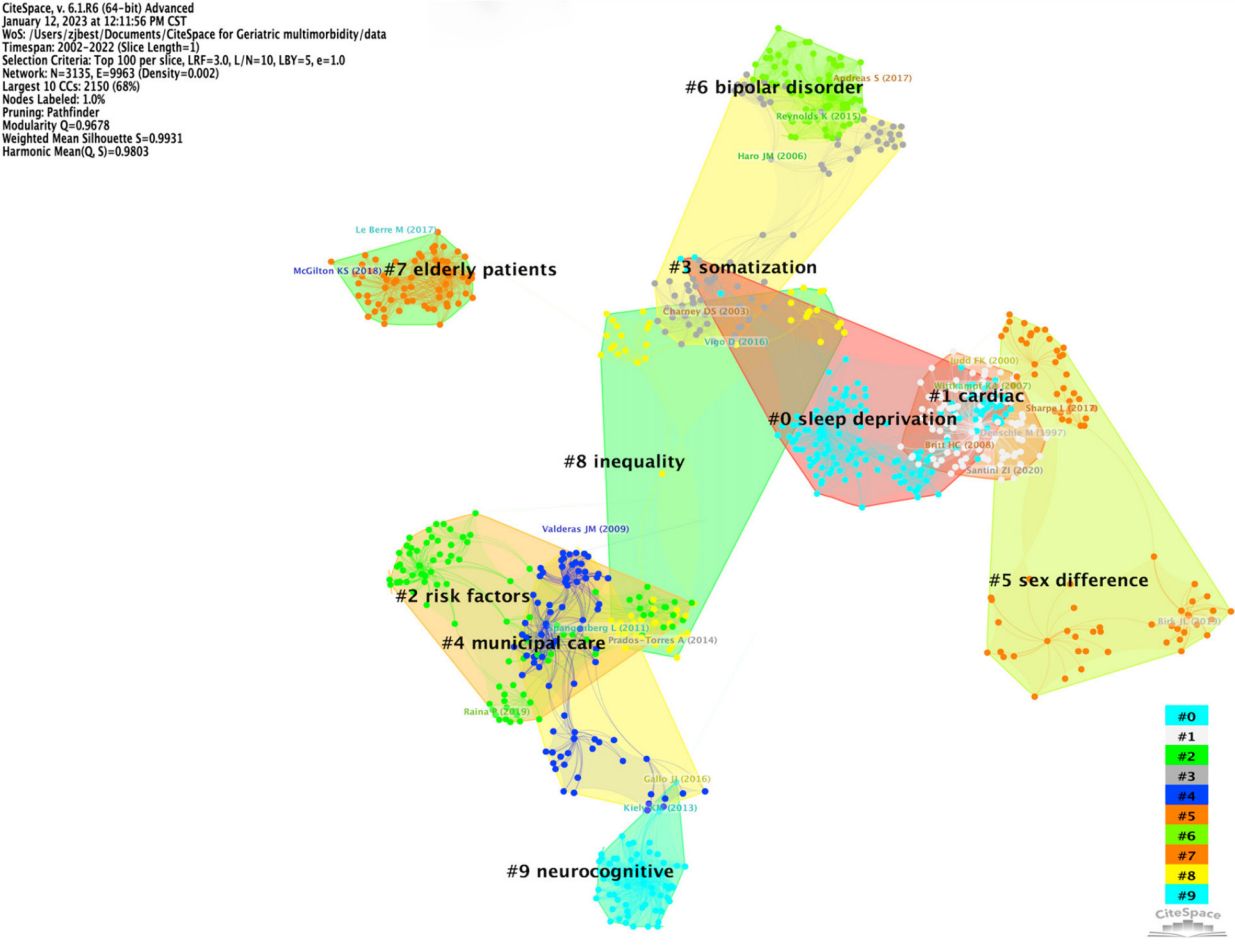
The clusters of the reference co-citation network.

Through visual analysis of the clusters and core nodes in a reference co-citation network, literature plays a critical role in the evaluation of the research field's frontier could be found (17). Figure 6 displays the top ten clusters of the reference co-citation network, of which the clusters tagged as “Sleep deprivation” (#0) ranked first place, followed by “Cardiac” (#1), “Risk factors” (#2), “Somatization” (#3), “Municipal care” (#4), “Sex difference” (#5), “Bipolar disorder” (#6), “Elderly patients” (#7), “Inequality” (#8), and “Neurocognitive” (#9). Research in the past two decades mainly focused on investigating the prevalence (sex difference, risk factors) and relevant impacts on elderly patients, such as psychical disorders (cardiac disorders, sleep deprivation, somatization) and mental health (bipolar disorder, neurocognitive). According to the network's modularity (Q = 0.9678) and silhouette (S = 0.9931), the findings of this knowledge map were considered valid and reliable.

The Top five cited references regarding frequency and centrality are presented in Table 5. By analyzing the ranking of cited references in frequency and centrality, most were review articles and observational research like cross-sectional and cohort studies. A few-second analysis of the national dataset was also detected. “Multimorbidity and depression: A systematic review and meta-analysis” (8) was the most frequently cited reference published in the *Journal of Affective Disorders*. This review aimed to explore the association between multimorbidity and depression.

**Table 5:** The top five cited references in terms of highest frequency and centrality.

**Ranking**	**Frequency**	**Cited reference**	**References**
1	19	Multimorbidity and depression: a systematic review and meta-analysis	Read et al. (8)
2	6	Epidemiology of multimorbidity and implications for health care, research, and medical education: a cross-sectional study	Barnett et al. (27)
3	6	Depression and multimorbidity: considering temporal characteristics of the associations between depression and multiple chronic diseases	Birk et al. (28)
4	5	Associations between somatic multimorbidity patterns and depression in a longitudinal cohort of middle-aged and older Chinese	Yao et al. (29)
5	5	Global multimorbidity patterns: a cross-sectional, population-based, multi-country study	Garin et al. (30)
**Ranking**	**Centrality**	**Cited reference**	**References**
1	0.23	Depression and multimorbidity: a cross-sectional study of 1,751,841 patients in primary care	Smith et al. (31)
2	0.18	Problem-solving therapy and supportive therapy in older adults with major depression and executive dysfunction: effect on disability	Alexopoulos et al. (32)
3	0.17	How does sex influence multimorbidity? secondary analysis of a large nationally representative dataset	Agur et al. (33)
4	0.14	Age-and gender-specific prevalence of depression in latest-life—Systematic review and meta-analysis	Luppa et al. (34)
5	0.14	Prevalence, determinants and patterns of multimorbidity in primary care: a systematic review of observational studies	Violan et al. (35)

Moreover, it highlighted that those with multimorbidity were twice to three times riskier of suffering from depression when compared to those without multimorbidity or no chronic physical disorders. In addition, a 45% greater risk was identified in people with multimorbidity for each additional chronic disorder than in those without any chronic physical disorders. As per the centrality, the study “Depression and multimorbidity: a cross-sectional study of 1,751,841 patients in primary care” (31) ranked top of the cited reference. This original article was a secondary analysis of data from 314 primary care practices in Scotland. Primary care depression was linked to a broad spectrum of physical comorbidities. Since the nature and extent of multimorbidity and its crucial relationship with social deprivation had not been explored based on a representative dataset of routine primary care data, the findings had significant implications for the integrated management of depression and multimorbidity in the United Kingdom and worldwide.

### Keywords analysis

**Figure 7:**
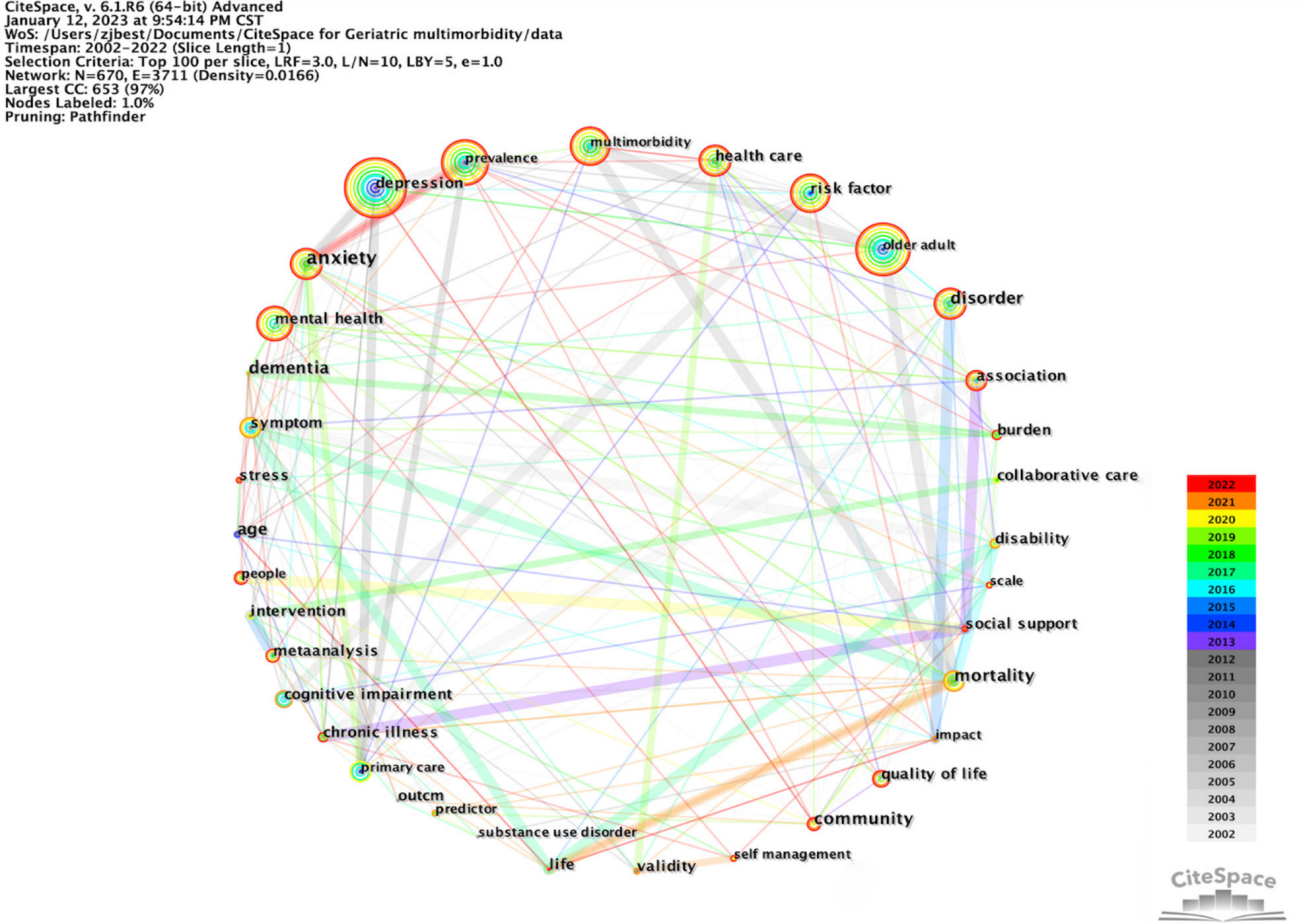
A keyword co-occurrence map.

Keyword co-occurrence refers to how frequently two keywords appear in the same citation. As Keywords present the essential feature of literature, analyzing keyword co-occurrence frequency and centrality can help to identify research hotspots (18, 26). Figure 7 shows the co-occurrence network of keywords, which consists of 670 nodes and 3,711 links based on a top *N* = 100% selection criteria. Similar keywords with the same meaning were merged. For instance, “old adults,” “old age,” “elderly people,” and “elderly patients” were merged as “old adult.” Moreover, The topic terms “old adult,” “multimorbidity,” “mental health,” “depression,” “disorder,” and “anxiety” were employed in the search strategy. This figure showed notable concerns for older adults with multimorbidity about the prevalence of mental disorders such as depression and anxiety. Furthermore, considerable research has focused on the relevant impact, like the quality of life and mortality (21, 36). Nevertheless, depression was the core topic in this research field of mental health and multimorbidity in older people. Besides, other types of mental disorders (anxiety) and psychiatric disorder (bipolar disorder) were also worthy of investment (20, 37). The network's keyword co-occurrence density (0.0166) indicated a weak relationship between keywords.

Table 6 lists the top ten keywords in terms of frequency and centrality. Apart from the search terms, the keyword with the highest frequency was “depression,” while “anxiety” ranked first regarding centrality. In addition, the top six keywords of the highest centrality shared reliable betweenness centrality, which was all over 0.1. Analyzation by comparing Figure 7 and Table 6 confirmed that depression and anxiety was the focal topic in the field.

**Table 6:** The top ten keywords of highest frequency and centrality.

**Ranking**	**Frequency**	**Keyword**	**Ranking**	**Centrality**	**Keyword**
1	81	Older adults	1	0.23	Anxiety
2	79	Depression	2	0.13	Age
3	67	Prevalence	3	0.12	Dementia
4	61	Multimorbidity	4	0.12	Disorder
5	49	Health care	5	0.11	Mortality
6	42	Risk factor	6	0.11	Healthcare
7	36	Mental health	7	0.08	Mental health
8	32	Disorder	8	0.07	Risk factor
9	27	Quality of life	9	0.07	Outcome
10	25	Anxiety	10	0.07	Heart failure

### Co-citation analysis of keywords

**Figure 8:**
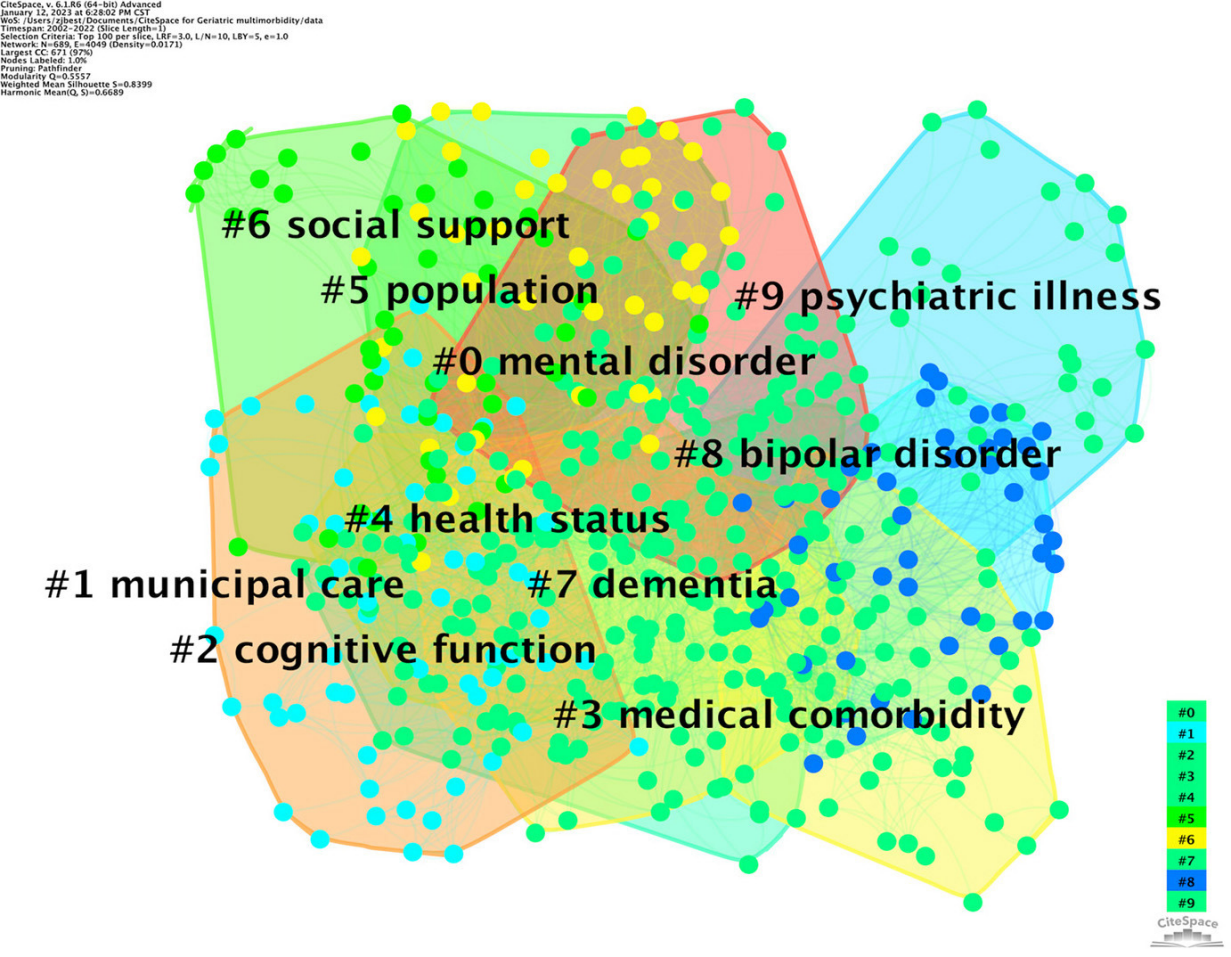
Clusters analysis of keyboard network.

Figure 8 presents the network of keywords co-occurrence by the log-likelihood ratio (LLR). “Mental disorders” (#0) was the largest cluster, followed by “municipal care” (#1), “cognitive function” (#2), “medical comorbidity” (#3), “health status” (#4), “population” (#5), “social support” (#6), “dementia” (#7), “bipolar disorder” (#8), and “psychiatric illness” (#9). The clusters of the co-occurrence of keywords in the past 20 years revealed that research on mental health and multimorbidity in older people mainly concentrated on mental disorders (#0, #2, #7, #8, #9), such as psychiatric illness and bipolar disorder. Meanwhile, continuous monitoring of the coexistence of multimorbidity and mental health (#3, #4, #5) and integrated interventions for prevention and management, such as municipal care and social support, were warranted (#1, #6). According to the network's modularity (Q > 0.3) and silhouette (S > 0.7), the findings of this knowledge map were regarded as robust and convincing.

### Keyword burst

**Figure 9:**
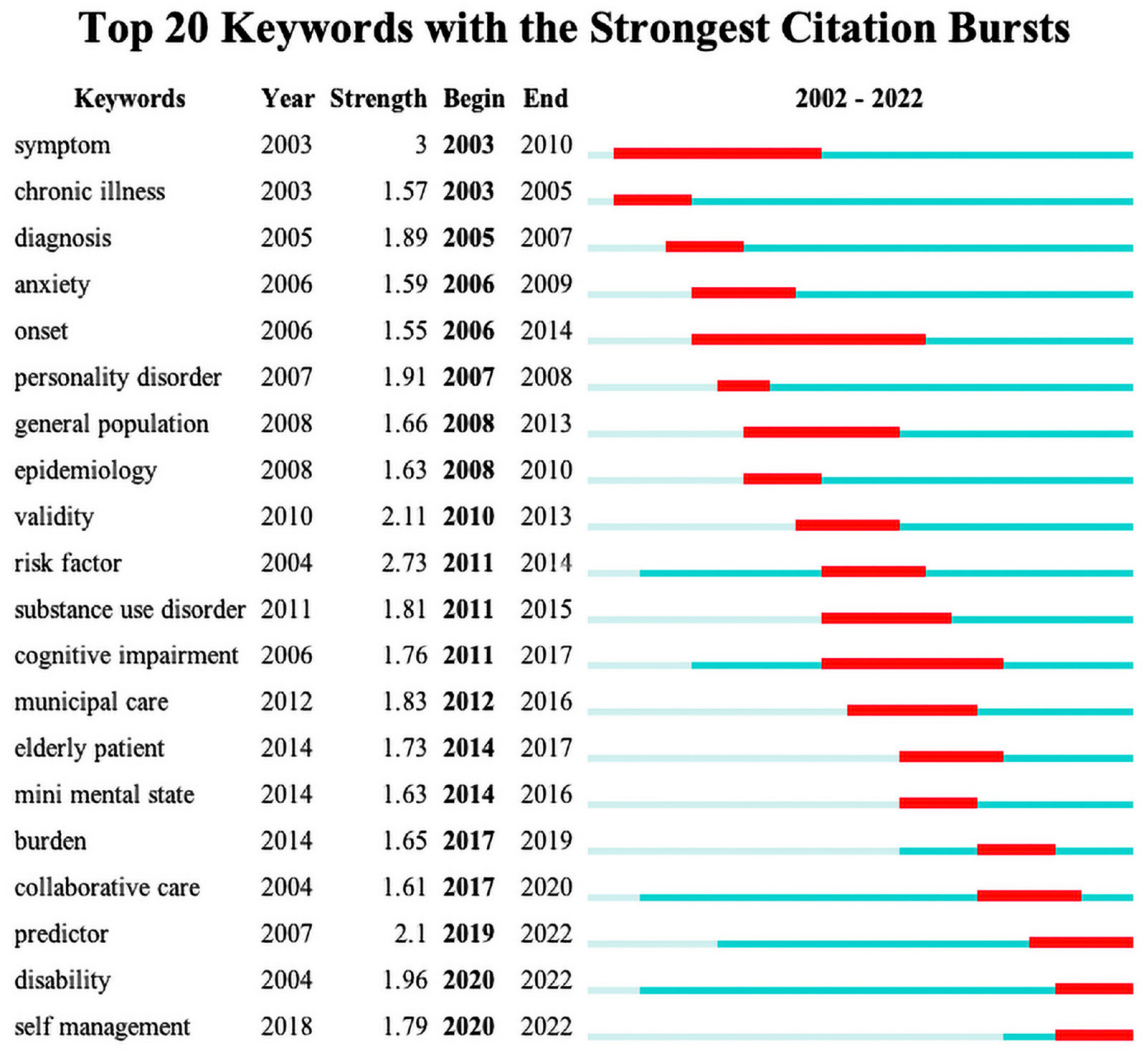
Top 20 keywords with the strongest citation burst.

Keyword burst is keywords cited frequently over a certain period. Analyzing keyword burst maps could assist in detecting frontiers and investigating the evolution of the research area (38, 39). Figure 9 depicted the top 20 keywords with the most vigorous citation burst between 2002 and 2022, of which the burst period was underlined in red. The figure identified no specific keyword in 2002, possibly due to the low volume of relevant publications. Then the remaining keywords bursts could be generally divided into three phases: (1) Emerging research interest had investigated the coexistence of mental disorders and multimorbidity, symptoms, and epidemiology were analyzed to support diagnosis (2003–2010). (2) Considerable studies focused on comprehensive evaluation and management, which shed light on risk factors and the validity of relevant assessment checklists and scales. In addition, multidimensional healthcare, like collaborative and municipal care, were also research hotspots during this period (2011–2019). (3) Given the critical impact on the quality of later life, especially disability, interventions on prevention and integrated management, including physical activity and self-management, were continuously explored (2020–2022).

## Discussion

### General information

This bibliometric analysis focused on the topic of mental health among the elderly with multimorbidity. Kuzuya (40) emphasized that multimorbidity in old patients has already become the era of geriatric medical challenges. Furthermore, previous studies revealed a bidirectional relationship between mental health and multimorbidity in old patients (41). For instance, a longitudinal cohort study reported that early-onset depression could raise the risk of future multimorbidity, and multimorbidity also contributed to an increased risk of depression in elderly Chinese adults (42). However, healthcare staff currently still practice “in the dark” due to the uncertainty of any effective interventions. Thus, a comprehensive overview of this research field is significantly urged for potential improvements.

CiteSpace-generated knowledge maps based on a rigorous literature search revealed that mental health and multimorbidity in older persons were a global concern. Developed countries dominated scientific research. The University of Pittsburgh, Stanford University, and the University of Michigan are recognized as the top three most important institutions in the United States. Likewise, the majority of representative researchers in the subject originated from industrialized nations. Despite the fact that each has built a highly regarded research unit, cooperation between these research forces are rather limited.

### Research themes analysis

By analyzing the results of co-citation reference clusters, co-occurrence network of keywords, co-citation keywords clusters, and keywords citation bursts, the research field on mental health and geriatric multimorbidity could be split into four themes.

1. Social psychology as the disciplinary foundation Dual-overlay of cited articles highlighted *Social Psychology* as the fundamental research discipline. Similar results were observed in the keyword co-occurrence network, where depression ranked second in frequency (79) and centrality (0.13). *Health, Nursing, Medicine* have major philosophical significance as well.

2. Prevalence of mental disorders and multimorbidity in old adults. Prior research and surveys focused mostly on the prevalence of elderly individuals with multimorbidity who later developed mental problems. Consequently, conceptional analysis of multimorbidity and mental health in older individuals has been investigated most frequently. Mental health and multimorbidity were associated in both directions as common risk factors in older persons.

3. Pertinent health conditions. Considering the limited examination of viable interventions for any changes, and the relatively subjective or superficial nature of earlier studies, the health status of the defined group have inevitably deteriorated. Meanwhile, extremely intense outpatient healthcare systems struggled with tremendous medical burdens and impending issues of considerably greater magnitude.

4. Effective interventions. Given continuous research evaluating prevention and treatment options, relevant research has focused on integrated geriatric management, which is primarily concerned with prevention and management. After a thorough evaluation of symptoms and risk factors, early expert diagnoses were obtained. Therefore, successful prevention could result in improved treatment. As per the management interventions, healthcare services and social support addressed integrated care. In coping with more complicated and advanced healthcare practices, integrated care was cited more often than collaborative and social care. In the meanwhile, it was felt that social assistance, such as neurotypical peer support and municipal care, merited additional investigation.

### Research trends analysis

As presented in the keywords burst map, *symptoms and onset* saw the most extended burst periods, indicating that a large amount of research was attracted to investigate the association between multimorbidity and mental health in older adults. Moreover, various types of mental disorders like *anxiety, personality disorder, cognitive impairment, and mini-mental state* were identified, highlighting mental health with the highest burst intensity. Recently, predictors for related prognoses like *disability* and effective interventions like *self-management* have become the present research trends. Furthermore, according to the co-occurrence network, *depression* ranked top of the most frequently cited keywords and was regarded as the current research hotspot.

Overall, publications on multimorbidity and mental health in old adults have expanded dramatically in the past 20 years and are likely to continue to be a popular research topic. Future research calls for worldwide cooperation among primary forces.

### Limitation

Additionally, this study has several limitations. Firstly, Due to the low likelihood of a publication eruption, the findings of this research are applicable under restrictions. Secondly, since the findings are mainly around developed countries, situations in developing countries may notably change. At last, CiteSpace is constantly updated, whereas personal learning of the application may be temporally limited. Nonetheless, this study provided reliable findings based on systematic search and objective data.

## Conclusion

We visually analyzed 216 Web of Science Core Collection records in this study using CiteSpace (6.1.R6 advanced). By adjusting various parameters, specific knowledge maps identified that effective intervention for improved prognosis had become the current research trend. Meanwhile, depression and anxiety were considered the hotspots in mental health and multimorbidity research in older adults. Concerning the bidirectional relationship between multimorbidity and mental health in old adults, promising exploration in this field could inspire more beneficial research forces on the particular aged adults. Thus, to make a step further substantial evidence-based prevention and treatment strategies are warranted for improved prognoses.

## Data availability statement

The original contributions presented in the study are included in the article/Supplementary material, further inquiries can be directed to the corresponding author.

## Author contributions

LZ and JZ designed this research. JZ drafted the manuscript. DS proofread the final draft and controlled the quality of the article. CW and QC conducted the acquisition and analysis of data. GZ and JM processed the figures and tables and interpretation. All authors contributed to the article and approved the submitted version.
