# Supplementary figures and images for: Research trends in the mental health and multimorbidity of older people from 2002 to 2022: A bibliometric analysis via CiteSpace
Source: Front Psychiatry. 2023 Mar 7;14:1117554. doi: 10.3389/fpsyt.2023.1117554 (PMC10027769; doi:10.3389/fpsyt.2023.1117554)

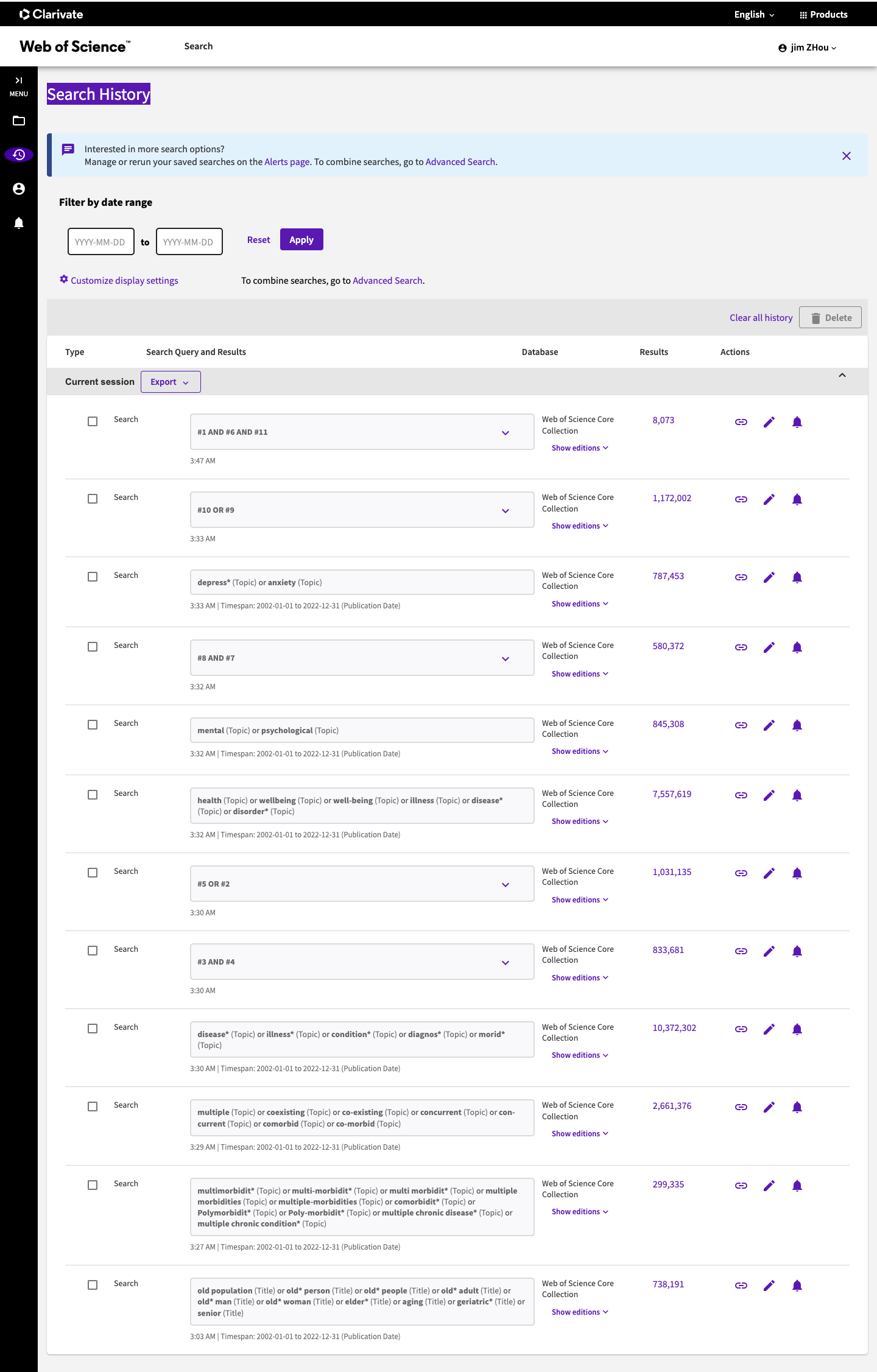

Supplement: Supplementary file 1 [file Image_1.TIF]
